# A Single Hot Event Stimulates Adult Performance but Reduces Egg Survival in the Oriental Fruit Moth, *Grapholitha molesta*

**DOI:** 10.1371/journal.pone.0116339

**Published:** 2014-12-31

**Authors:** Li-Na Liang, Wei Zhang, Gang Ma, Ary A. Hoffmann, Chun-Sen Ma

**Affiliations:** 1 Climate Change Biology Research Group, State Key Laboratory for Biology of Plant Diseases and Insect Pests, Institute of Plant Protection, Chinese Academy of Agricultural Sciences, Beijing, China; 2 Pest and Environmental Adaptation Research Group, Departments of Zoology and Genetics, Bio21 Institute, The University of Melbourne, Victoria, Australia; Federal University of Viçosa, Brazil

## Abstract

Climate warming is expected to increase the exposure of insects to hot events (involving a few hours at extreme high temperatures). These events are unlikely to cause widespread mortality but may modify population dynamics via impacting life history traits such as adult fecundity and egg hatching. These effects and their potential impact on population predictions are still largely unknown. In this study, we simulated a single hot event (maximum of 38°C lasting for 4 h) of a magnitude increasingly found under field conditions and examined its effect in the oriental fruit moth, *Grapholitha molesta*. This hot event had no impact on the survival of *G. molesta* adults, copulation periods or male longevity. However, the event increased female lifespan and the length of the oviposition period, leading to a potential increase in lifetime fecundity and suggesting hormesis. In contrast, exposure of males to this event markedly reduced the net reproductive value. Male heat treatment delayed the onset of oviposition in the females they mated with, as well as causing a decrease in the duration of oviposition period and lifetime fecundity. Both male and female stress also reduced egg hatch. Our findings of hormetic effects on female performance but concurrent detrimental effects on egg hatch suggest that hot events have unpredictable consequences on the population dynamics of this pest species with implications for likely effects associated with climate warming.

## Introduction

Global climate change is expected to not only lead to a substantial increase in average temperature but also in the frequency of extreme hot events [1]. Daily maximum temperatures often climb to high levels in summer in many orchards and particularly in exposed places such as the canopy of fruit trees [2]. Here, high temperatures are being increasingly recorded, as illustrated by the incidence of periods>38°C in two locations in China where fruit production carried out (S1 Fig.). The impact of such occasional hot events on pest and beneficial invertebrates have rarely been considered, particularly because 1) the extreme high temperatures often last for few hours in a single day and do not necessarily affect survival [3], and 2) the hot events tend not to increase average temperatures experienced across an entire generation of invertebrates, and average temperatures are popularly used in population prediction [4], [5].

While high temperatures can impact the population dynamics of insects in the field [6]–[8], laboratory studies of such effects typically involve constant high temperatures [9]–[11], fixed temperature cycles [12]–[14] or more rarely repeated heat stress [15]–[17] that do not necessarily capture short extreme conditions. Some studies have considered the relationship between extreme heat stress and adult survival, by defining critical thermal maxima [18]–[20] and upper lethal temperatures [21]–[23]. However the results of these studies can be hard to relate to the field where thermal maxima might not be experienced. In contrast, there is limited information on how rare but non-lethal high temperatures affect life history traits and mating ability, which could both influence the dynamics of insect populations.

High temperatures can lead to negative or positive effect on the performance of adult insects. On the one hand, high temperature for few hours can reduce longevity and fecundity in species including *Trialeurodes vaporariorum* and *Bemisia tabaci* [24], *Metopolophium dirhodum* [15] and *Helicoverpa armigera* [25], reduce fecundity and egg mass in *Bicyclus anynana* [26], and decrease mating success in *H. armigera* [25] and *Drosophila* [27], [28]. On the other hand, adult performance measures such as longevity and heat tolerance can also be increased through mild heat stress, and this phenomenon is termed heat-induced hormesis [29]–[32]. Hormesis may potentially increase reproductive output which can be correlated with extended longevity [33], [34]. Such apparent hormetic effects are known for other stressors such as a low dose of toxins [35]–[39]. In addition, high temperature exposure can impact offspring performance through parental effects [40], [41].

In a previous study, we found that a single hot event can reduce hatched eggs but had no detrimental effects on immediate adult performance in the diamondback moth *Plutella xylostella* [3]. *P. xylostella* are exposed their whole lives to ambient fluctuating temperatures and have high heat resistance [42], [43]. Here we test for detrimental effects and hormetic effects of a single hot event on one of the most important pests on fruit trees worldwide, the oriental fruit moth, *Grapholitha molesta*. While immature stages of this species are to some extent buffered from thermal extremes (larvae are found in fruit or twig tissues and pupae are found in bark), adults are exposed to ambient conditions when they seek mates and resources. Thus, the adults may be vulnerable to encountered hot events. We therefore considered (1) how a single hot event imposed on adults affected adult performance; (2) whether any effects were sex specific, and (3) whether effects were carried over through the egg stage, resulting in parental effects. To address these questions, females or males were exposed to hot events and tested for survival, longevity, and copulation, while females were tested for oviposition responses. Effects on egg hatch were also considered.

## Materials and Methods

### Insect rearing

Laboratory populations were established in 2008 by the Department of Plant Protection, Northwest Agricultural and Forestry University in China. The colony was maintained at 25±1°C, 60–80% RH and 15L: 9D. For maintaining the colony, new-born larvae were reared firstly on apples until these rotted, and then were transferred to artificial diet until pupation. The components of the artificial diet were prepared as described in Du [44].

For the experiments, about 50 black-headed eggs were placed on the stalk cavity of a Fuji apple. Newly hatched larvae bored into the apple to feed and develop. Apples were held in a plastic box (20×10×10 cm) to reduce dehydration. When apples began to rot, larvae were transferred to a glass tube (1 cm diameter ×10 cm long: 5 larvae per tube) in which 3 g artificial diet had been placed. These tubes were covered with cotton gauze to prevent escape, and to provide a pupation surface. Pupae were moved to glass jars (15 cm diameter ×20 cm) for emergence. Fifty pairs of male and female moths emerging within 24 h were introduced to a plastic container (15×35 cm) for copulation and oviposition. Adults were provided with a cotton ball with 10% honey solution for feeding. Females oviposited on the surface of the plastic container. After oviposition, the container was cut up to provide samples of around 50 eggs. Eggs were sprayed with water daily until the head of embryo became black, and were then placed on the stalk cavity of a Fuji apple for the ensuing rearing cycle. Newly emerged females and males from this generation were used in experiments, after they were fed for three days in glass tubes (1 cm diameter ×10 cm long, closed with a cotton ball containing 10% honey solution).

### Experimental design and methods

To assess the impacts of heat stress on the performance of each sex, four treatments were set up: (1) the control group in which both sexes were kept at 25°C with 60–80% RH (female/male: 25/25); (2) males exposed to 38°C with 60–80% RH but females which remained at 25°C (25/38); (3) females exposed to the heat stress but males that remained at 25°C (38/25); (4) both sexes exposed to 38°C (38/38). 62 males and 31 females were tested for each treatment. Stressed females or males in glass tubes were directly immersed into an ethylene glycol bath at 38°C for 4 h (Ministat 230-cc-NR, Huber GmbH, Germany). Non-stressed females or males were kept at 25°C for 4 h in the rearing room. Adult survival was then recorded immediately by checking movement of the body and any appendages. All moths survived heat exposure.

For each treatment, 31 females were each exposed to two males in glass tubes (5 cm diameter ×10 cm long). The duration of copulation of each female was monitored for four hours. Then, all tested pairs were reared and allowed to copulate and oviposit at 25°C with 60–80% RH until all adults died. Survival and egg production in the tubes were checked daily. Moths were transferred daily to new glass tubes, so that the number of hatched eggs could also be recorded.

### Statistical analysis

To analyze the effect of heat exposure on the survival of females and males, we used a Cox proportional hazard model in the package “survival” in R, with female and male temperature as fixed factors. For the other traits, we first tested whether variables were normally distributed by a Shapiro-Wilk test in SPSS software (IBM SPSS Statistics 20, IBM Corporation, Somers, NY, USA). We defined the pre-copulation period as the duration before the first copulation after pairing, the copulation period as the duration of the first copulation, and the pre-oviposition period as a period between adult pairing and the onset of reproduction. The oviposition period represented the number of days from the beginning to the end of oviposition, while the post-oviposition period was the number of days between females ceasing reproduction and dying. The pre-copulation period, copulation period, lifetime fecundity, oviposition period, ln-transformed post-oviposition period and square-root arcsine-transformed egg hatching success followed normal distributions; two-way ANOVAs were therefore run on these variables with female and male temperature treatment as fixed factors (PROC GLM, SAS V9.2). Data of pre-oviposition period followed a Poisson distribution, and treatment effects were therefore examined by using a generalized linear model with female and male temperature treatment as fixed factors (PROC GENMOD, Poisson errors, logit link; SAS V9.2). There was no significant interaction between female and male temperatures, so we dropped interactions from the final analysis.

Eggs were not produced beyond 30 days after heat treatments. To examine the persistent effects of treatments on egg hatching success, the variances of hatching rate across treatments every three days across the 30 day period were analyzed with Kruskal-Wallis tests using IBM SPSS Statistics. To visualize changes across time, we plotted changes in mean egg hatching success. To test if female reproductive performance was associated with delayed oviposition, we analyzed the relationships between pre-oviposition period and lifetime fecundity/oviposition period by fitting a linear regression model. Because of heteroscedasticity, we ran regressions on reciprocally transformed variables as well as untransformed variables, and we also checked significance of regression coefficients using bootstrapping. These analyses were run in IBM SPSS Statistics.

To determine overall fitness effects from the female or/and male heat treatments, we estimated the net reproductive value as:



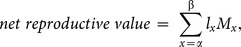
where x is age in days, α is the age at start of reproduction, β is the age at the end of reproduction, *l_x_* is the proportion of individuals being alive at age *x*, and *M_x_* is the average number of offspring (hatched eggs) produced by living females age *x*.

## Results

### Immediate mortality and longevity

**Figure 1:**
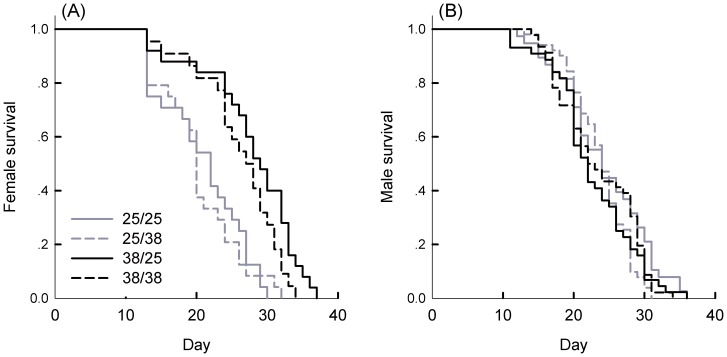
Survival curves of females and males in *G. molesta* after different temperature treatments. Treatments involved different combinations of female and male exposure to either 25°C or 38°C for 4 h. 25/25: both males and females were kept at 25°C; 25/38: females were remained at 25°C but males were stressed at 38°C; 38/25: females were stressed at 38°C and males were remained at 25°C; 38/38: both females and males were stressed at 38°C.

Heat exposure at 38°C for 4 h did not cause immediate mortality of *G. molesta* adults, but survival of females declined at a slower rate over time when females were stressed (Z = −2.171, df = 1, 93; P = 0.03; Fig. 1, A). As a consequence, the mean longevity of stressed females was 27.1 d, which was 6.4 d longer than for non-stressed females. The survival curve of males was not affected by heat treatments (Z = 0.97, df = 3, 179; P = 0.33), with a mean (±SD) longevity of 23.5±5.5 d (Fig. 1, B).

### Mating behavior

**Figure 2:**
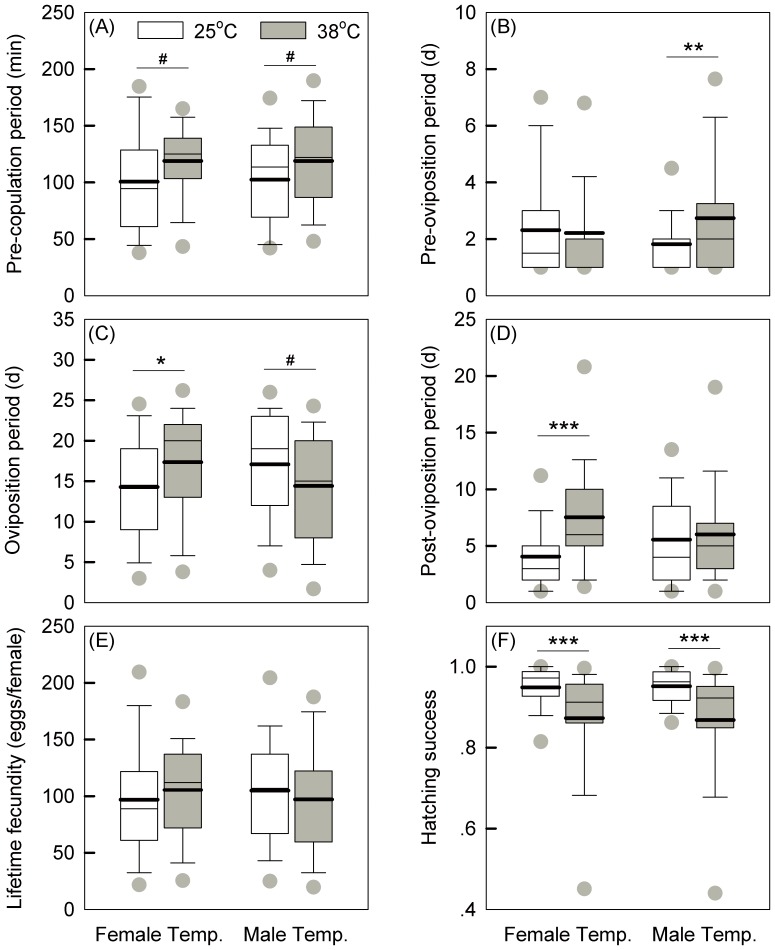
Sex effects on reproduction traits after heat exposure. Treatments involved female or male treatment either at 25°C or 38°C for 4 h. The thin and thick horizontal lines within the boxes represent medians and means respectively. The asterisks (*) and octothorpes (#) indicate the significant differences between female or male heat treatments at different levels (^#^ *P*<0.1; *P<0.05; **P<0.01, ***P<0.001).

Heat stress did not influence the copulation period, with an average time of 28±8 (SD) min (Table 1). Female or male heat exposure led to a prolonged pre-copulation period, extending this by around 18 min and 16 min respectively (Fig. 2, A); these differences were borderline non- significant (Table 1).

**Table 1:** Results of general linear models testing the effects of female and male temperature (25°C versus 38°C) on reproduction traits.

Source	df	F (or Chi-Square)	P
Pre-copulation period (min)			
Female temperature	1	3.91	0.059
Male temperature	1	3.10	0.083
Error	69		
			
Copulation period (min)			
Female temperature	1	1.12	0.296
Male temperature	1	1.74	0.192
Error	56		
Pre-oviposition period (d)^a^			
Female temperature	1	0.05	0.820
Male temperature	1	8.90	**0.003**
Error	92		
Oviposition period (d)			
Female temperature	1	4.80	**0.031**
Male temperature	1	3.56	0.062
Error	92		
Post oviposition period (d)			
Female temperature	1	20.17	**<0.0001**
Male temperature	1	0.90	0.346
Error	92		
Lifetime fecundity (eggs/female)			
Female temperature	1	0.74	0.391
Male temperature	1	0.61	0.437
Error	92		
Egg hatching success			
Female temperature	1	22.12	**<0.0001**
Male temperature	1	24.46	**<0.0001**
Error	92		

Significant p-values are given in bold.

a The significance of pre-oviposition period were examined by a generalized linear model under a Poisson distribution using the chi-square value.

### Periods of pre-oviposition, oviposition and post-oviposition

Females that copulated with stressed males had an extended pre-oviposition period, on average 0.9 day longer than for unstressed males (Table 1; Fig. 2, B). Female heat exposure led to a marginally significant increase in oviposition period which was extended by more than two days (Table 1; Fig. 2, C); females mated with males exposed to heat showed a shorter oviposition period, although the treatment effect was marginally non-significant (Table 1; Fig. 2, C). Stressed females had almost double the post-oviposition period than unstressed females, and this difference was highly significant (Table 1; Fig. 2, D).

### Fecundity and egg hatching

**Figure 3:**
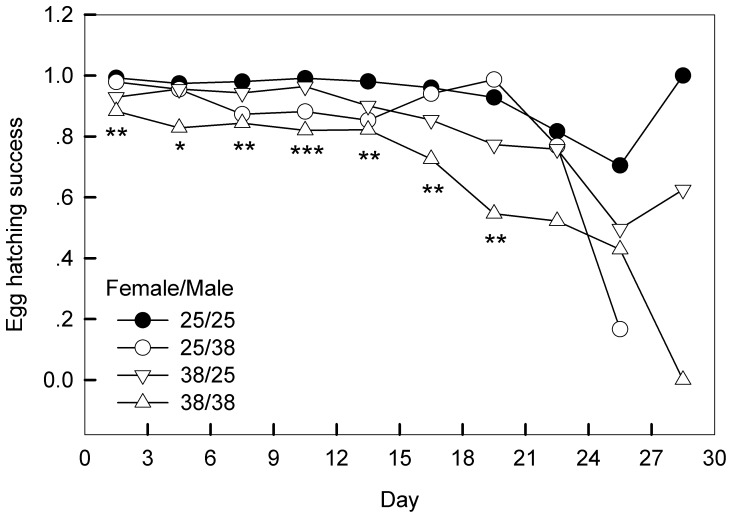
Impact of heat stress on egg hatching success every three days across the whole oviposition period. Treatments involve different combinations of female and male treatment either at 25°C or 38°C for 4 h. 25/25: both males and females were kept at 25°C; 25/38: females were remained at 25°C but males stressed at 38°C; 38/25: females were stressed at 38°C and males were remained at 25°C; 38/38: females and males were stressed at 38°C. The asterisks below each plot indicate significant differences between treatments (*P<0.05; **P<0.01, ***P<0.001).

Female heat exposure led to an 8.9% increase in lifetime fecundity, whereas male heat exposure led to a 7.5% decrease in fecundity (Fig. 2, E), although these differences in lifetime fecundity were not significantly affected either by female or male treatment (Table 1). Egg hatching success significantly decreased when either of the sexes was exposed to heat stress (Table 1; Fig. 2, F). The control group (25/25) had the highest hatching rate (0.98±0.10), followed by treatments involving heat stress only on females (38/25: 0.93±0.11) or males (25/38: 0.92±0.13). When stressed females were mated with stressed males (38/38), hatching success was at its lowest level (0.81±0.22). During the entire oviposition period, heat treatments reduced daily egg hatching success (Fig. 3), reflecting persistent effects on this trait.

**Figure 4:**
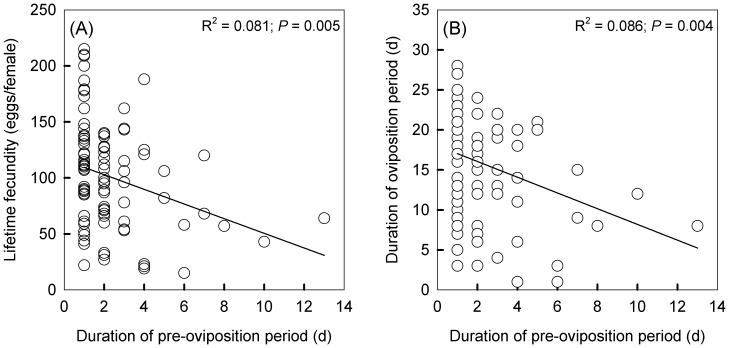
Relationships between pre-oviposition period and lifetime fecundity or oviposition period. The linear regression analysis showed that delayed oviposition after heat exposures had minor impacts on the lifetime fecundity (y = 116–6.5x, R^2^ = 0.081, *P* = 0.005) and duration of oviposition period (y = 18.0–1.0x, R^2^ = 0.086, *P* = 0.004).

We analyzed the relationships between pre-oviposition period and lifetime fecundity/oviposition period. The regression results showed that delayed oviposition after heat exposures had a highly significant impact on lifetime fecundity (y = 116–6.5x, R^2^ = 0.081, P = 0.005; Fig. 4, A) and duration of oviposition period (y = 18.0–1.0x, R^2^ = 0.086, P = 0.004; Fig. 4, B). These relationships remained significant when we computed significance based on regressions with reciprocally transformed dependent variables to reduce heteroscedasticity (lifetime fecundity: P = 0.009; duration of oviposition: P = 0.019) and also when significance of the regression coefficients was tested through bootstrapping (lifetime fecundity: P = 0.002; duration of oviposition: P = 0.016). To assess the overall fitness effect from female and/or male heat exposure, we examined the net reproductive value. The results showed that there was a decline in net reproductive value when males were exposed to heat (female/male, 25/38: 86.6; 38/38: 87.2) compared to the control treatment (25/25: 98.8), but there was no substantial difference when females from the control treatment were compared to those exposed to heat (38/25: 101.0).

## Discussion

Hot days with a daily maximum temperature of ≥38°C for several hours are common in the main production area of pome fruit trees in China and also in other parts of the world. Here we investigated the impacts of a single hot event on the performance of *G. molesta* adults and the consequences for egg hatching success of the next generation. Our results indicated that a single hot event (38°C for 4 h) has no significant effects on the survival of *G. molesta* adults, copulation period or male longevity. However, mating behavior was transiently inhibited after female or male heat stress. Imposing heat either on males or females reduced egg hatching success significantly. The sexes showed different responses to the stress periods. Exposed females had a longer lifespan and oviposition period and perhaps an increase in lifetime fecundity. However, heat exposure in males delayed the onset of oviposition of females, as well as reducing the duration of oviposition period and lifetime fecundity. Overall, male heat exposure markedly reduced the net reproductive value of *G. molesta*. These findings, combined with the fact that a single hot event changed the temporal pattern of daily egg hatching, suggest that a single event can modify the dynamics of *G. molesta* populations.

### Female longevity and hormesis

Our results indicate that a single hot event extended longevity and may also have increased lifetime fecundity. Short periods of exposure to high temperature can induce a hormetic response that leads to a prolonged life span in insects [29]–[32], and many insects show a positive relationship between lifetime fecundity and longevity [33], [34]. However, the effect we detected was relatively small, particularly when compared to inter-individual variability in lifetime fecundity which can vary over a wide range in insects [33], [45], [46]. Small changes in lifetime fecundity under stress may also occur in other insects such as *H. armigera* [25], *Plutella xylostella* [3] and *Drosophila* [47]. An increase in fecundity has also been found in insects exposed to a low dose insecticide [35]–[39].

These hormetic effects at the adult stage were not carried over to their offspring because a single hot event decreased egg hatching success (Fig. 2, F), either in the early or late phase of egg laying (Fig. 3). Such a reduction in egg hatching success following adult heat exposure has been found in other insect species [3], [48], and might be explained by the ‘dynamic energy budget’ hypothesis [49], [50]. Lepidoptera like *G. molesta*, do not take in nitrogen sources during adult stage, but allocate limited nitrogen resources to maintain adult life or to form mature eggs. Stressful conditions often stimulate adults to lay eggs as early as possible to avoid the possible detrimental impacts in their late life period [51], [52]. *Grapholitha molesta* has an intermittent egg-laying pattern in which females require a period between oviposition bouts, and under heat stress perhaps inadequately resourced eggs were laid.

### Different effects on males or females

While heat stress resulted in some positive effects on females, heat stress had negative effects on males expressed indirectly as an extended pre-oviposition period (Fig. 2, B). This effect may reflect delayed mating by stressed males [27], [28], [53], which can lead to a decline in female reproduction in *G. molesta* [54], [55]. The longer pre-oviposition period was also associated somewhat with a reduction in female fecundity and duration of the oviposition period (Fig. 4). Heat stress in males reduce the proportion of reproductive females in a population [56] and even their mean fecundity [53]. Although the mechanism underlying the effects we detected is unclear, there may be a reduction in the resources that males pass to females during mating, such as a reduction in functional sperm [47] or in male ejaculate which can supply nutrition to females [57], [58].

### Conclusions

While a hot day may not change the average temperature much or increase the number of degree days much for development, it may have an impact on population performance even when conditions do not exceed the CTmax of adults. By altering the timing of oviposition and abundance of adults, eggs and hatchlings through improving female survival and perhaps their lifetime fecundity as well as reducing hatching success, the local pest pressures exerted by *G. molesta* may change. Under climate change, the frequency of extreme hot days is expected to increase [1], and species like *G. molesta* with multiple generations a year face an increased likelihood of experiencing hot events across summer. While detrimental effects of hot days have been noticed by ecologists [3], potential hormetic effects and their consequences have not been considered in population dynamics. The hormetic effects on adult performance caused by a single hot event may result in an increase in population density. However, this increase does not persist because of a decline in offspring survival. It remains to be seen if such effects can be detected under field conditions and whether multiple hot days result in population increases or the collapse of pest populations.

## Supporting Information

S1 Fig **Hot days (DTmax ≥38°C) in June-August at Baoding and Zhengzhou from 1980–2014**. Anomalies in the number of hot days (to the average form 1980–2014) at Baoding (A) and Zhengzhou (B). Daily maximum temperature records were downloaded from the China Meteorological Data Sharing Service System.
(TIF)
